# Supplementary material for: Age influences on the molecular presentation of tumours
Source: Nat Commun. 2022 Jan 11;13:208. doi: 10.1038/s41467-021-27889-y (PMC8752853; doi:10.1038/s41467-021-27889-y)
Supplement: Supplementary file 10 — Reporting Summary [file 41467_2021_27889_MOESM10_ESM.pdf]

nature research

Corresponding author(s): Paul C Boutros

Last updated by author(s): Dec 16, 2021

Reporting Summary

Nature Research wishes to improve the reproducibility of the work that we publish. This form provides structure for consistency and transparency in reporting. For further information on Nature Research policies, see our Editorial Policies and the Editorial Policy Checklist.

Statistics

For all statistical analyses, confirm that the following items are present in the figure legend, table legend, main text, or Methods section.

n/a	Confirmed	
☐	☒	The exact sample size (*n*) for each experimental group/condition, given as a discrete number and unit of measurement
☐	☒	A statement on whether measurements were taken from distinct samples or whether the same sample was measured repeatedly
☐	☒	The statistical test(s) used AND whether they are one- or two-sided<br>*Only common tests should be described solely by name; describe more complex techniques in the Methods section.*
☐	☒	A description of all covariates tested
☐	☒	A description of any assumptions or corrections, such as tests of normality and adjustment for multiple comparisons
☐	☒	A full description of the statistical parameters including central tendency (e.g. means) or other basic estimates (e.g. regression coefficient) AND variation (e.g. standard deviation) or associated estimates of uncertainty (e.g. confidence intervals)
☐	☒	For null hypothesis testing, the test statistic (e.g. *F*, *t*, *r*) with confidence intervals, effect sizes, degrees of freedom and *P* value noted<br>*Give P values as exact values whenever suitable.*
☒	☐	For Bayesian analysis, information on the choice of priors and Markov chain Monte Carlo settings
☒	☐	For hierarchical and complex designs, identification of the appropriate level for tests and full reporting of outcomes
☐	☒	Estimates of effect sizes (e.g. Cohen's *d*, Pearson's *r*), indicating how they were calculated

Our web collection on statistics for biologists contains articles on many of the points above.

Software and code

Policy information about availability of computer code

Data collection	Publicly available data was downloaded from Broad GDAC Firehose (TCGA), Synapse (PCAWG), and the GDC Data Portal (AACR-GENIE). No additional software was used
Data analysis	All statistical analyses and data visualization were performed in the R statistical environment (v3.2.1) using the BPG (v5.9.8) and Survival (v2.44-1.1) packages, and with Inkscape (v0.92.3). Mutation sets generated by the TCGA, PCAWG and AACR-GENIE projects included the use of GISTIC 2.0 and MutSig v2.0.

For manuscripts utilizing custom algorithms or software that are central to the research but not yet described in published literature, software must be made available to editors and reviewers. We strongly encourage code deposition in a community repository (e.g. GitHub). See the Nature Research guidelines for submitting code & software for further information.

Data

Policy information about availability of data

All manuscripts must include a data availability statement. This statement should provide the following information, where applicable:

- Accession codes, unique identifiers, or web links for publicly available datasets
- A list of figures that have associated raw data
- A description of any restrictions on data availability

The Cancer Genome Atlas datasets were downloaded from Broad GDAC Firehose (https://gdac.broadinstitute.org/), release 2016-01-28. Open access TCGA data was used in this study.
Pan-cancer Analysis of Whole Genomes whole genome sequencing molecular profiles can be downloaded from the PCAWG consortium through the ICGC Data Portal at [https://dcc.icgc.org/releases/PCAWG]: consensus SNV and indels [https://dcc.icgc.org/releases/PCAWG/consensus_snv_indel], consensus copy number data [https://dcc.icgc.org/releases/PCAWG/consensus_cnv], subclonal reconstruction https://dcc.icgc.org/releases/PCAWG/subcloncal_reconstruction], and

mutational signatures data [https://dcc.icgc.org/releases/PCAWG/mutational_signatures] are available alongside clinical and histology annotation [https://dcc.icgc.org/releases/PCAWG/clinical_and_histology]. PCAWG data is controlled access and administered by dbGaP and ICGC Data Access Compliance Office. Information on accessing the data, including raw read files, can be found at [https://docs.icgc.org/pcawg/data/]. In accordance with the data access policies of the ICGC and TCGA projects, most molecular, clinical and specimen data are in an open tier that do not require access approval. To access potentially identification information, such as germline alleles and underlying sequencing data, researchers will need to apply to the TCGA Data Access Committee (DAC) via dbGaP for access to the TCGA portion of the dataset, and to the ICGC Data Access Compliance Office (DACO) for the ICGC portion. To access somatic single nucleotide variants derived from TCGA donors, researchers will also need to obtain dbGaP authorisation. Researchers may apply for access at [https://docs.icgc.org/download/data-access/].
Controlled access AACR GENIE data was downloaded from AACR Project GENIE for the MSK project91. [https://portal.gdc.cancer.gov/projects/GENIE-MSK]. Researchers may apply for access to AACR GENIE from dbGaP at [https://gdc.cancer.gov/access-data].

Field-specific reporting

Please select the one below that is the best fit for your research. If you are not sure, read the appropriate sections before making your selection.

☒ Life sciences ☐ Behavioural & social sciences ☐ Ecological, evolutionary & environmental sciences

For a reference copy of the document with all sections, see nature.com/documents/nr-reporting-summary-flat.pdf

Life sciences study design

All studies must disclose on these points even when the disclosure is negative.

Sample size

All data available from the TCGA, PCAWG, and AACR GENIE MSK projects were used with exclusions as described below. Cancer types with small sample sizes were ignored in tumour type-specific analyses. These datasets contain high quality mutation calls and sufficient annotation data for multivariable adjustment. They also span multiple tumour-types and together allow comparison of findings across three distinct cohorts.

Data exclusions

Data was excluded if annotation data describing age was unavailable, as this information is critical to the study design. Metastatic tumours from AACR GENIE MSK were also excluded to better match the primary tumours of TCGA and PCAWG

Replication

We performed our analyses in TCGA and PCAWG data independently using the same statistical framework. Results from these analyses overlap in some cases as described in the manuscript. We further added analysis of AACR-GENIE data as an additional validation set. Differing results may be due to intrinsic differences in the datasets including geographic origin of patients and data collection strategies. Results also did not replicate in tumour-types with small sample sizes. Results for all three cohorts are presented in the study and highlighted when findings are consistent across multiple studies.

Randomization

Not relevant as this study is an overview of age-associated differences in cancer genomics. All data used in study was previously collected by others. Response to treatment was not examined and randomization was not necessary.

Blinding

We did not study treatment response and blinding was unnecessary.

Reporting for specific materials, systems and methods

We require information from authors about some types of materials, experimental systems and methods used in many studies. Here, indicate whether each material, system or method listed is relevant to your study. If you are not sure if a list item applies to your research, read the appropriate section before selecting a response.

Materials & experimental systems

n/a	Involved in the study	
☒	☐	Antibodies
☒	☐	Eukaryotic cell lines
☒	☐	Palaeontology and archaeology
☒	☐	Animals and other organisms
☒	☐	Human research participants
☒	☐	Clinical data
☒	☐	Dual use research of concern

Methods

n/a	Involved in the study	
☒	☐	ChIP-seq
☒	☐	Flow cytometry
☒	☐	MRI-based neuroimaging
